# Distinct fingerprints of tRNA-derived small non-coding RNA in animal models of neurodegeneration

**DOI:** 10.1242/dmm.050870

**Published:** 2024-11-18

**Authors:** Sharada Baindoor, Hesham A. Y. Gibriel, Morten T. Venø, Junyi Su, Elena Perez Morrissey, Elisabeth Jirström, Ina Woods, Aidan Kenny, Mariana Alves, Luise Halang, Paola Fabbrizio, Maria Bilen, Tobias Engel, Marion C. Hogg, Caterina Bendotti, Giovanni Nardo, Ruth S. Slack, Jørgen Kjems, Jochen H. M. Prehn

**Affiliations:** ^1^ Department of Physiology and Medical Physics, Royal College of Surgeons in Ireland, 123 St Stephen's Green, Dublin D02 YN77, Ireland; ^2^ FutureNeuro SFI Research Centre for Chronic and Rare Neurological Diseases, Royal College of Surgeons in Ireland, Dublin D02 YN77, Ireland; ^3^ Omiics ApS, DK-8200 Aarhus N, Denmark; ^4^ Interdisciplinary Nanoscience Centre, Department of Molecular Biology and Genetics, Aarhus University, DK-8000 Aarhus C, Denmark; ^5^ Laboratory of Neurobiology and Preclinical Therapeutics, Department of Neuroscience, IRCCS - Mario Negri Institute for Pharmacological Research, Via Mario Negri, 2, 20156 Milan, Italy; ^6^ Department of Cellular and Molecular Medicine, University of Ottawa, Ottawa, ON K1H 8M5, Canada; ^7^ Department of Biosciences, Nottingham Trent University, Nottingham NG11 8NS, UK

**Keywords:** Amyotrophic lateral sclerosis, Frontotemporal dementia, Parkinson's disease, tsRNA, tRF, tiRNA

## Abstract

Transfer RNA-derived small RNAs (tsRNAs) – categorized as tRNA-derived fragments (tRFs), tRNA-derived stress-induced RNAs (tiRNAs) and internal tRF (itRF) – are small non-coding RNAs that participate in various cellular processes such as translation inhibition and responses to cellular stress. We here identified tsRNA profiles within susceptible tissues in animal models of amyotrophic lateral sclerosis (ALS), frontotemporal dementia (FTD) and Parkinson's disease (PD) to pinpoint disease-specific tsRNAs and those shared across neurodegenerative diseases. We performed small RNA sequencing in the SOD1^G93A^ and TDP43^A315T^ mouse models of ALS (spinal cord), the Tau^P301S^ model of FTD (hippocampus), and the parkin/POLG model of PD (substantia nigra). Bioinformatic analysis showed higher expression of 5′ tiRNAs selectively in the two ALS models, lower expression of 3′ tRFs in both the ALS and FTD mouse models, and lower expression of itRF Arg in the PD model. Experimental validation confirmed the expression of tsRNAs. Gene Ontology analysis of targets associated with validated 3′ tRFs indicated functions in the regulation of synaptic and neuronal pathways. Our profiling of tsRNAs indicates disease-specific fingerprints in animal models of neurodegeneration, which require validation in human disease.

## Teaser

**Summary:** Amyotrophic lateral sclerosis, frontotemporal dementia and Parkinson's disease are distinct neurological disorders with shared genetic mutations and pathologies. Here, we identified disease-specific and overlapping transfer RNA-derived small RNAs.

## INTRODUCTION

Neurodegenerative disorders (NDs) are distinguished by the deterioration of susceptible neurons in the brain, spinal cord or peripheral neurons. Age is an important risk factor for most NDs, along with exposure to genetic susceptibility and environmental factors (Patten et al., 2022; Nabi and Tabassum, 2022). Some of the major NDs include amyotrophic lateral sclerosis (ALS), frontotemporal dementia (FTD) and Parkinson's disease (PD).

ALS is a fatal ND caused by progressive loss of upper and lower motor neurons. ALS has a global incidence rate of 2 per 100,000 persons/year and a prevalence of 6 to 9 per 100,000 persons (Mead et al., 2023). Up to 90% of the cases are sporadic with no known causes, and 10% of cases are linked to specific gene mutations including chromosome 9 open reading frame 72 (*C9orf72*) repeat expansions, superoxide dismutase type 1 (*SOD1*), transactive response DNA-binding protein 43 (*TDP43*) and fused in sarcoma (*FUS*) mutations (Van Daele et al., 2023). Although the loss of motor neurons causes muscle weakness and wasting, it is typically the failure of the respiratory system that results in death within 2-5 years after the onset of the disease. ALS has a highly heterogeneous disease progression rate with a median of ∼3 years.

FTD is caused by the degeneration of neurons in the frontal and temporal lobes of the brain. This leads to a change in the behaviour, personality, language and cognitive capabilities of the affected individual. The estimated prevalence of FTD ranges from 0.01 to 4.61 per 1000 individuals, with an annual addition of 0.01 to 2.5 new cases per 1000 people (Leroy et al., 2021). Approximately 30% have a genetic form of FTD, with mutations mainly in *C9orf72*, progranulin (*GRN*) and microtubule associated protein tau (*MAPT*) genes (Greaves and Rohrer, 2019). FTD has a very heterogeneous survival rate, with the mean ranging from 2.5 to 8 years (Kansal et al., 2016).

PD is one of the most common NDs, with an estimated incidence of 13.43 in 100,000 persons/year and a prevalence of 106.28 in 100,000 persons (Ou et al., 2021). PD results from the degeneration of dopaminergic neurons in the substantia nigra pars compacta area of the brain. Affected individuals exhibit both motor and non-motor symptoms. Motor symptoms of PD include rigidity, imbalance and tremors, and non-motor symptoms include cognitive impairment, dementia and sleep disorders. Up to 95% of PD cases are sporadic and 5% are genetic, with mutations mainly in alpha synuclein (*SNCA*), *PARK2* (parkin), *PARK7*, PTEN induced kinase-1 (*PINK1*) and leucine-rich repeat kinase2 (*LRRK2*) (Tang et al., 2017).

Interestingly, there is an elevated risk of developing ALS, PD and FTD among individuals who are blood relatives of patients with familial ALS (Majoor-Krakauer et al., 1994; Fallis and Hardiman, 2009). About 15% of patients with ALS are also diagnosed with FTD (Masrori and Van Damme, 2020), and there is an extensive overlap of mutations shared between ALS and FTD, including *C9orf72* repeat expansions and *TDP43* mutations (Abramzon et al., 2020). ALS mutations shared between ALS and PD include mutations in angiogenin (*ANG*) (van Es et al., 2011). Additionally, these conditions manifest similar pathological features, including the presence of abnormal protein aggregates and neuroinflammation. Collectively, these studies suggest the existence of common disease pathways among ALS, FTD and PD.

With the ageing population on the rise and no cure available, it is imperative to work towards the development of diagnostic biomarkers that can facilitate earlier detection and intervention. Furthermore, given that ALS diagnosis relies on exclusionary methods, it becomes essential to identify a biomarker capable of distinguishing ALS from other NDs. Small non-coding RNAs (sncRNAs) have emerged as promising biomarkers for the diagnosis and prognosis of NDs (Watson et al., 2019). sncRNAs range from 18 to 200 nucleotides (nt) in length and encompass various types such as microRNA (miRNA), ribosomal RNA (rRNA), transfer RNA (tRNA), small nuclear RNA (snRNA), small nucleolar RNA (snoRNA) and piwi-interacting RNA (piRNA). tRNAs transport amino acids to the ribosome, which then adds the amino acids to the growing polypeptide chain during translation. They typically measure between 73 and 90 nt in length and exhibit a cloverleaf secondary and an L-shaped tertiary structure. tRNAs are cleaved under cellular stress conditions to form tRNA-derived small RNAs (tsRNA). tsRNAs can be further classified based on the cleavage site and size of the resulting fragments. tRNA cleaved in the anticodon loop by ANG generates tRNA-derived stress-induced RNAs (tiRNA) that are 30-40 nt in length (Yamasaki et al., 2009). Cleavage in the T/D loop by DICER or other RNases results in the formation of tRNA-derived fragments (tRFs), which are 14-30 nt in length. When tsRNAs are cleaved at both the T and D loop, an internal tRF (itRF) is formed. tsRNAs are recognized to have active roles in stress signalling and post-transcriptional regulation, such as messenger RNA (mRNA) silencing, translational repression and inhibition of apoptosis (Kuhle et al., 2023). Although selective tsRNAs have been shown to be generated in neurological disorders, the complete scope of tsRNAs and their functions in neurons remain to be fully elucidated (Fagan et al., 2021).

In this study, we aimed to illustrate the spectrum of tsRNA fragments in disease-relevant areas of ALS, FTD and PD to identify the fragments that are generated owing to common neurodegenerative processes or that are specific to these individual diseases and would allow us to differentiate the diseases from each other.

## RESULTS

### Abundance of tsRNAs and reduced presence of miRNAs in hippocampus and substantia nigra relative to spinal cord

**Fig. 1.**
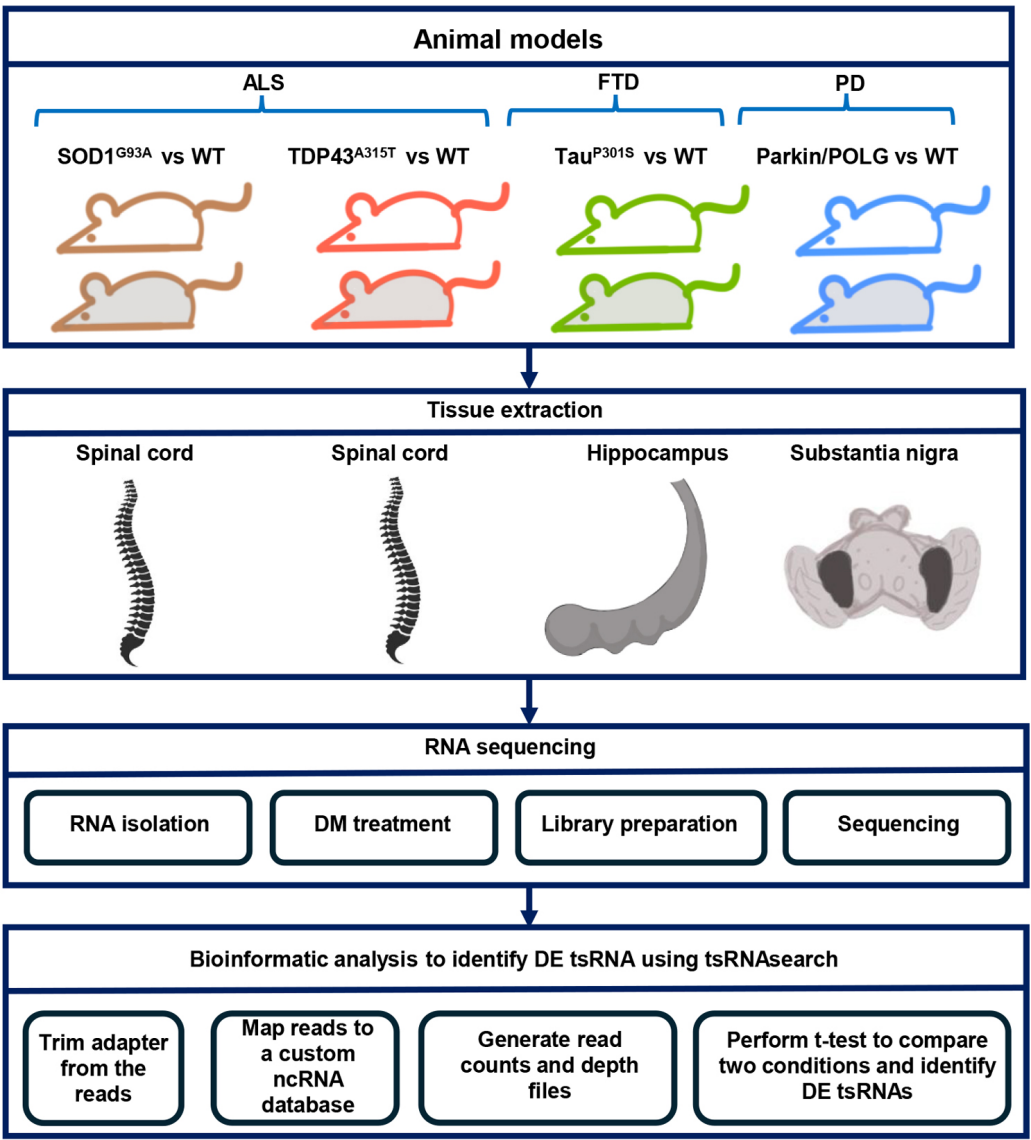
**Study design.** The figure shows the animal models and tissues used, and sequencing steps and bioinformatic analysis conducted to identify differentially expressed transfer RNA-derived small RNAs (tsRNAs). ALS, amyotrophic lateral sclerosis; DE, differentially expressed; DM, demthylation; FTD, frontotemporal dementia; ncRNA, non-coding RNA; PD, Parkinson's disease; WT, wild type.

**Fig. 2.**
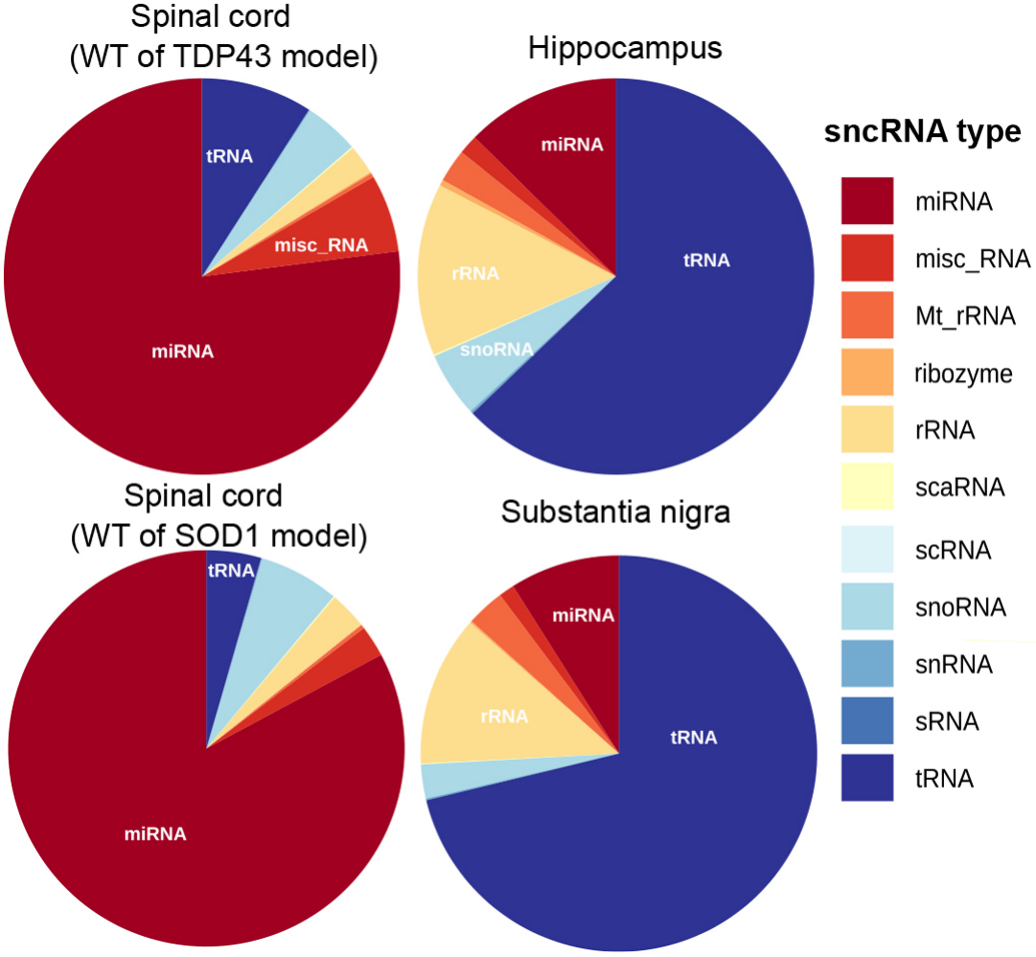
**sncRNA composition in different tissues.** Pie charts depicting the different classes of small non-coding RNA (sncRNA). The colours represent different sncRNA classes in spinal cord from WT of TDP43 (*n*=4) and SOD1 (*n*=4) samples, hippocampus from Tau (*n*=3) samples, and substantia nigra from parkin/POLG (*n*=3) samples. miRNA, microRNA; misc_RNA, miscellaneous RNA; Mt_rRNA, mitochondrially encoded rRNA; rRNA, ribosomal RNA; scaRNA, small Cajal body-specific RNA; scRNA, small cytoplasmic RNA; snoRNA, small nucleolar RNA; snRNA, small nuclear RNA; sRNA, small RNA; tRNA, transfer RNA.

We performed small non-coding RNA-sequencing (RNA-seq) analysis of SOD1^G93A^ and TDP43^A315T^ models of ALS (spinal cord), a Tau^P301S^ model of FTD (hippocampus) and a parkin/POLG model of PD (substantia nigra), as well as their respective non-transgenic controls (Fig. 1). We observed that ∼86%, ∼70%, ∼11% and ∼9% of the total reads were from miRNAs in the spinal cord (SOD1), spinal cord (TDP43), hippocampus and substantia nigra of non-transgenic samples, respectively. tRNAs were the second major class of small non-coding RNA identified in non-transgenic samples and constituted ∼5%, ∼12%, ∼68% and ∼73% of the total reads in SOD1, TDP43, Tau and parkin/POLG samples, respectively. rRNAs made up approximately 2%, 3%, 12% and 11% of the reads, respectively. Less than 5% of the reads belonged of the reads belonging to snoRNA class (Fig. 2). Interestingly, we noted a similar composition in the corresponding mutants, suggesting that tissue origin rather than disease processes was the primary determinant of the composition of sncRNAs (Fig. S1).

### Length distribution profile of tsRNAs shows the presence of tiRNAs only in the SOD1 and TDP43 models of ALS

**Fig. 3.**
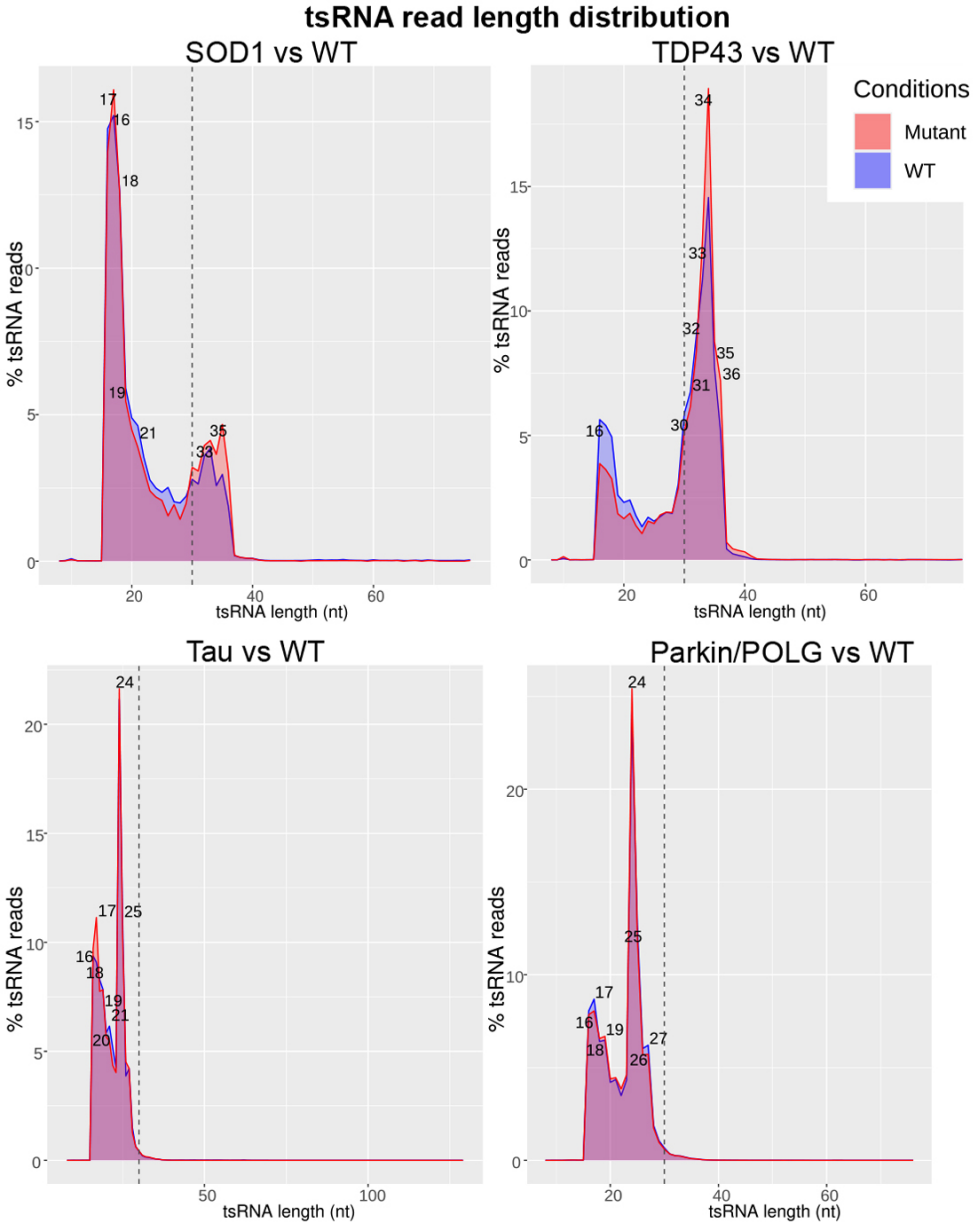
**tsRNA read length distribution.** tsRNA read lengths in the SOD1 (*n*=4 in mutant and WT), TDP43 (*n*=4 in mutant and WT), Tau (*n*=4 in mutant and *n*=3 in WT) and parkin/POLG (*n*=2 in mutant and *n*=3 in WT) models. The *x*-axis indicates the length of tsRNA in nucleotides (nt); the *y*-axis shows the percentage of tsRNAs. The vertical line at position 30 of the *x*-axis separates the tRFs on the left from tiRNAs on the right. Red, mutant; blue, WT.

**Fig. 4.**
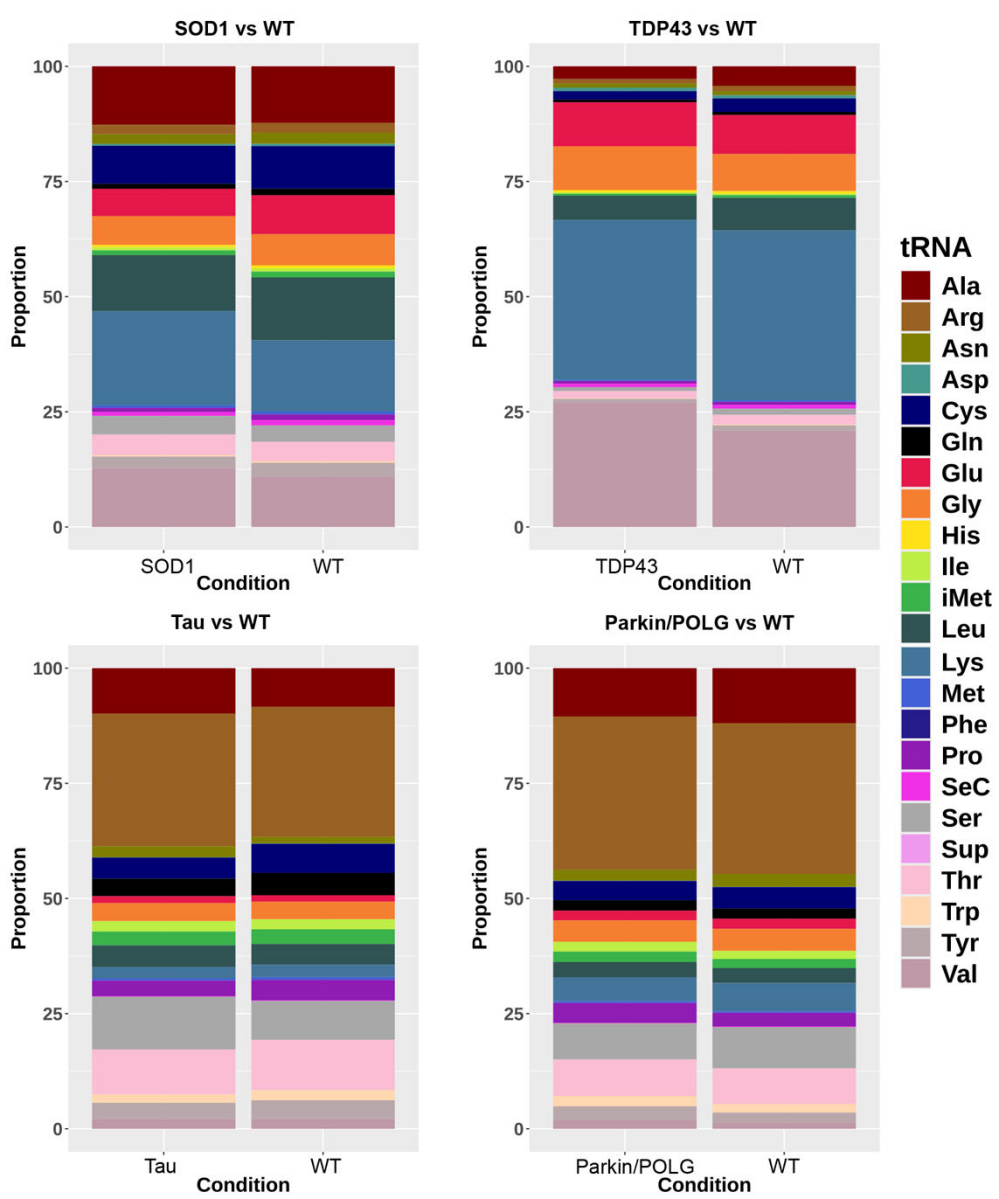
**tsRNA species in WT.** Stacked bar plots showing tRNA species identified in WT animals in the study. The *x*-axis shows the conditions SOD1, TDP43, Tau, parkin/POLG and their respective WT; the *y*-axis shows the proportion of reads that belong to each tRNA species. The colours indicate the tRNA species. Ala, alanine; Arg, arginine; Asn, asparagine; Asp, aspartic acid; Cys, cysteine; Gln, glutamine; Glu, glutamic acid; Gly, glycine; His, histidine; Ile, isoleucine; iMet, initiator methionine tRNA; Leu, leucine; Lys, lysine; Met, methionine; Phe, phenylalanine; Pro, proline; SeC, selenocysteine; Ser, serine; Sup, suppressor; Thr, threonine; Trp, tryptophan; Tyr, tyrosine; Val, valine.

We aimed to categorize the various classes of tsRNA present in our data and examine the similarities and differences across ALS, FTD and PD. We used tsRNAsearch (Donovan et al., 2021) to analyse the lengths of tsRNA fragments identified in this study. This revealed the presence of tiRNAs of length 30-36 bp in the two ALS models only and tRFs of length 17-27 bp in all four animal models of the three NDs (Fig. 3). We used tRAX (Holmes et al., 2022 preprint) to further verify the tsRNA expression profile in the animal models. The results from tRAX were consistent with the results from tsRNAsearch, indicating that only the ALS animal model showed the presence of 5′ tiRNAs, while both the ALS and FTD models consistently revealed the presence of 3′ tRFs (Fig. S2) in the transgenic models compared to the non-transgenic controls. Further analysis showed the prominence of tsRNAs from lysine, leucine, alanine and valine in the SOD1 mutant and respective non-transgenic controls, constituting approximately 58% and 52% of all tsRNA reads. In the TDP43 model, tsRNAs from lysine, valine, glutamic acid and glycine contributed approximately 80% and 74% of all tsRNA reads in the mutant and its respective non-transgenic controls. In the Tau and parkin/POLG models, arginine, serine, threonine and alanine formed more than 50% of the total tsRNA reads in mutants and their respective non-transgenic controls (Fig. 4). We checked the presence of amino acids arginine, serine, threonine and alanine in the proteins in the hippocampus and substantia nigra (midbrain) of the mouse genome (UniProt protein ID UP000000589). These four amino acids constitute 26.5% of the amino acids in proteins. Although this may suggest a higher demand for their respective tRNAs, it does not fully explain the increased presence of tsRNAs.

### Reduced expression of 3′ tRFs observed in the ALS and FTD mouse models

**Fig. 5.**
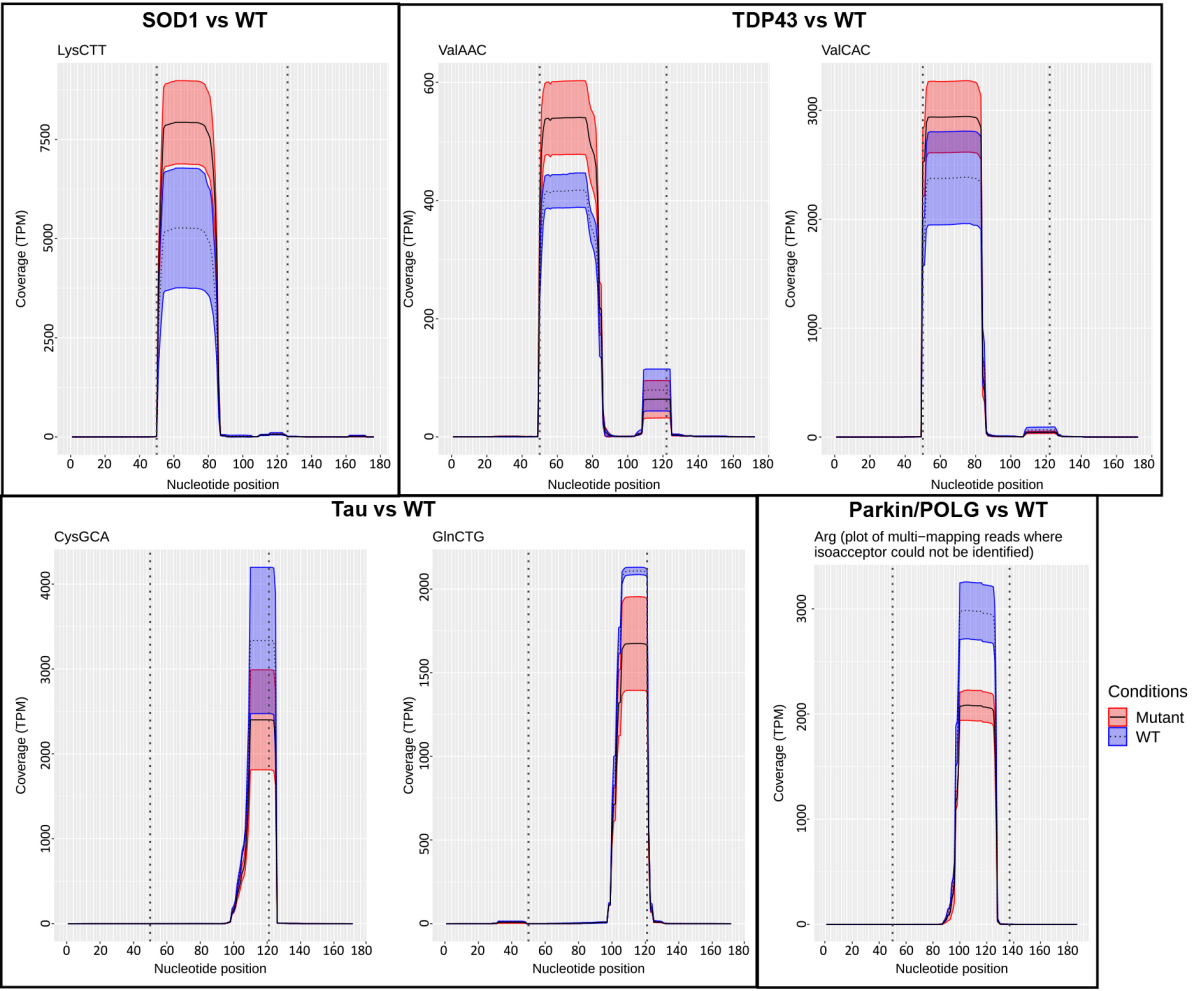
**Coverage plots of differentially expressed tsRNAs.** Coverage plots of 5′ tRNA-derived stress-induced RNA (tiRNA) LysCTT in SOD1 (*n*=4) and its WT (*n*=4), 5′ tiRNA ValAAC and 5′ tiRNA ValCAC in TDP43 (*n*=4) and its WT (*n*=4), 3′ tRNA-derived fragment (tRF) CysGCA and 3′ tRF GlnCTG in Tau (*n*=4) and its WT (*n*=3), and internal tRF (itRF) Arg in parkin/POLG (*n*=2) and its WT (*n*=3). The *x*-axis represents the nucleotide position; the *y*-axis represents the coverage in transcripts per million (TPM). The two vertical dotted lines enclose the main tRNA segment of 70 nt, with an additional 50 nt included both upstream and downstream. The dotted line indicates the mean coverage for WT samples; the solid line represents the mean coverage for the mutant conditions SOD1, TDP43, Tau and parkin/POLG; the shaded areas indicate the s.d. for both conditions.

**Fig. 6.**
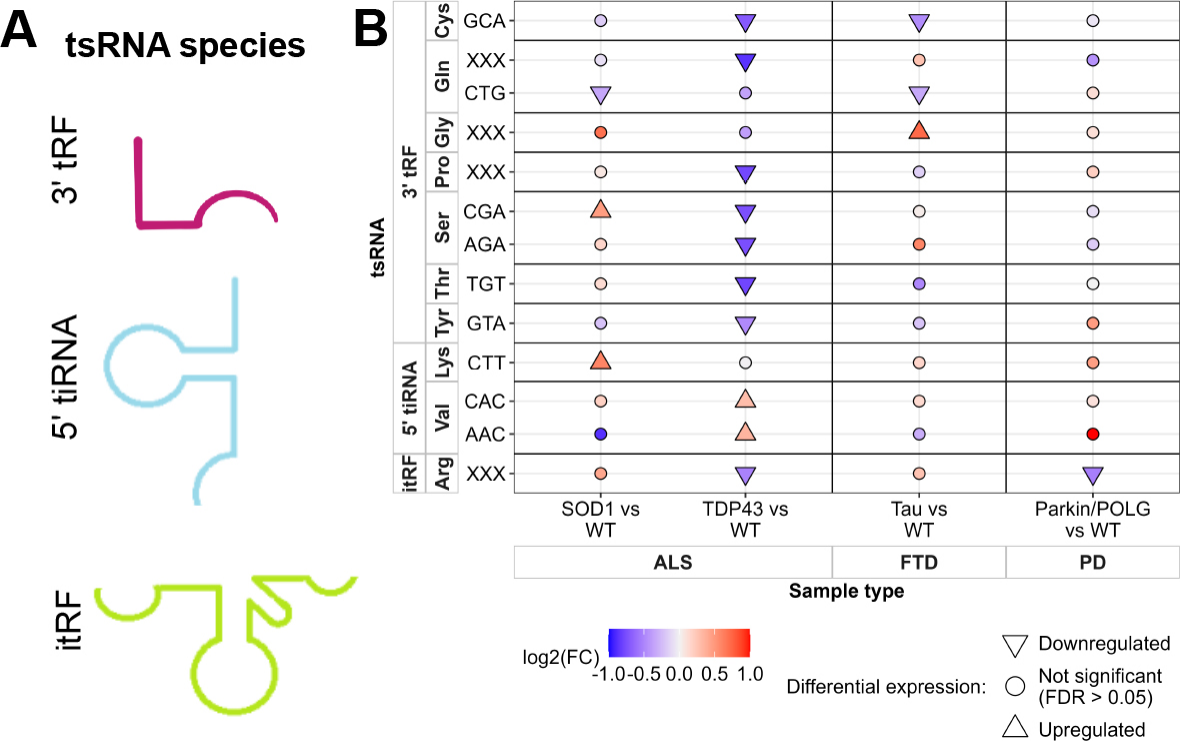
**Summary of differentially expressed tsRNAs.** (A) Secondary structure of the different tsRNA fragments identified in the study. (B) Summary of differentially expressed tsRNAs from the SOD1 (*n*=4) versus WT (*n*=4), TDP43 (*n*=4) versus WT (*n*=4), Tau (*n*=4) versus WT (*n*=3), and parkin/POLG (*n*=2) versus WT (*n*=3) comparison representing amyotrophic lateral sclerosis (ALS), frontotemporal dementia (FTD) and Parkinson's disease (PD). The *x*-axis shows the comparison and the *y*-axis shows the tsRNA. The shape represents the regulation: upward-facing arrowhead for upregulated, downward-facing arrowhead for downregulated and circle for not significant. The colour indicates the log2 fold change (log2FC). FDR, false discovery rate.

Next, we identified the tsRNAs that were differentially expressed between the mutant and their respective non-transgenic controls in this study. We employed tsRNAsearch to identify tsRNAs (Table S2) and plot the coverage of differentially expressed tsRNA fragments (Fig. 5). The coverage plot shows that the expression of ANG-derived tsRNA, 5′ tiRNA LysCTT, was upregulated in the mutant SOD1 mice, and 5′ tiRNA ValCAC and 5′ tiRNA ValAAC were upregulated in the mutant TDP43 mice, compared to their respective wild type (WT). We observed dysregulation of 3′ tRFs in the two ALS and FTD mouse models. In the SOD1 mouse model of ALS, we observed upregulation of 3′ tRF SerCGA in the mutant compared to the WT. In the TDP43 mouse model of ALS, we observed lower expression of seven 3′ tRFs in the mutant compared to the WT (additional coverage plots are shown in Fig. S3). In the FTD mouse model, we observed lower expression of 3′ tRF CysGCA and 3′ tRF GlnCTG in the mutant compared to the WT. In this mouse model, 3′ tRF Gly had higher expression in the Tau mutant compared to the WT. Interestingly, in the PD mouse model, we also observed lower expression of itRF Arg in the parkin/POLG mutant compared to the non-transgenic control. Fig. 6A shows the structure of different tsRNA fragments identified in the study. To determine the differentially expressed tsRNAs in the three animal models, a fold change (FC) cut-off value of more than 0.8 and less than 1.2, and a false discovery rate (FDR) cut-off of 0.05, was set (Fig. 6B). The TDP43 mouse model showed upregulation of 5′ tiRNA ValCAC (FC=1.23, log2FC=0.30 and FDR=2.982×10^−08^) and 5′ tiRNA ValAAC (FC=1.27, log2FC=0.34 and FDR=1.0974×10^−06^) in the mutant compared to the WT. Higher expression of 5′ tiRNA LysCTT in the SOD1 mouse model, as well as 5′ tiRNA ValCAC and 5′ tiRNA ValAAC in the TDP43 mouse model formed by ANG cleavage at the anticodon loop, suggests higher ANG activity in the mutant of the ALS mouse models. Lower expression of 3′ tRFs (except 3′ tRF SerCGA in the SOD1 ALS model and 3′ tRF Gly in the FTD model), formed by cleavage in the T loop of the tRNA, in the ALS and FTD animal models suggests lower activity of DICER or other RNases in the mutant than in WT.

### Validation of 3′ tRFs in the mouse models using Arraystar qPCR arrays

**Fig. 7.**
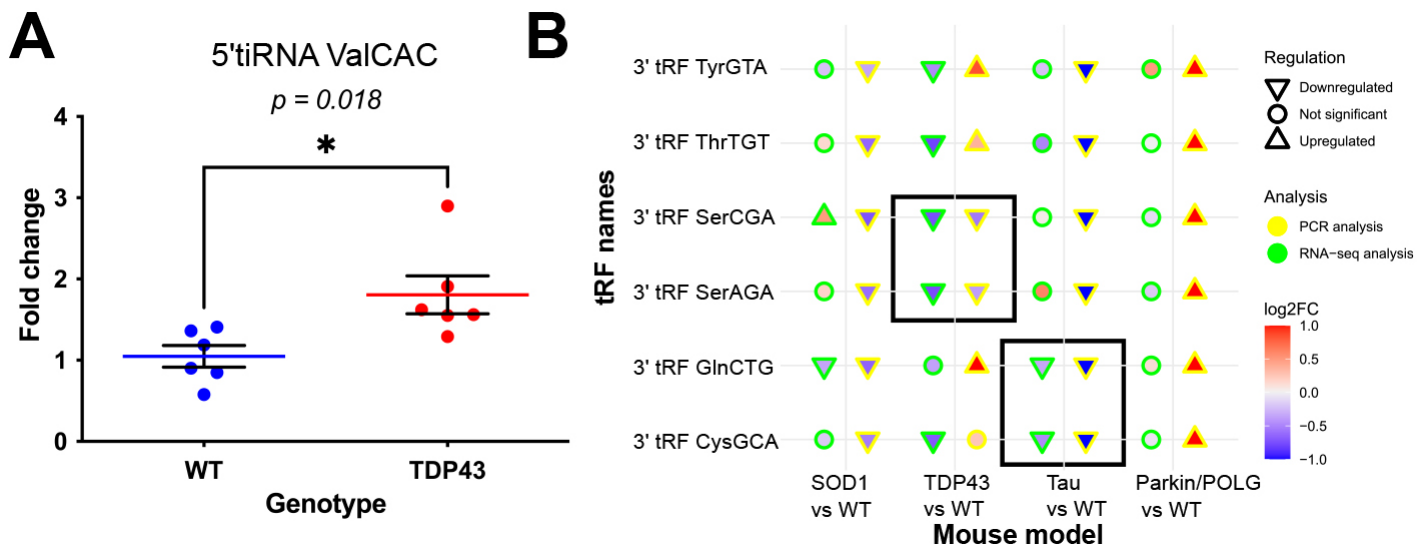
**qPCR validation of tsRNAs.** (A) Levels of 5′ tiRNA ValCAC in TDP43 and its WT quantified using a custom Taqman assay. Unpaired two-tailed Student's *t*-test, *P=0.018; individual data points are represented by the blue and red dots for WT and TDP43 mutant, respectively (*n*=6 individual data points in each condition). The bars show mean±s.d. (B) Plot comparing the small RNA-sequencing (RNA-seq) and quantitative PCR (qPCR) results. The *x*-axis shows the comparisons and the *y*-axis shows 3′ tRFs. The shape represents the regulation: upward-facing arrowhead for upregulated, downward-facing arrowhead for downregulated and circle for not significant. The colour indicates the log2FC. The colour of the border of the shape indicates the analysis type: yellow for PCR and green for small RNA-seq analysis. Significant results that match in both small RNA-seq and PCR analysis are enclosed within a black rectangular box.

We previously showed elevated levels of 5′ tiRNA ValCAC in the spinal cords of slow-progressing compared to fast-progressing SOD1 mice by TaqMan analysis using primers that specifically detect 5′ ValCAC tiRNA fragment (Hogg et al., 2020). The same custom TaqMan assays were utilized to quantify the levels of 5′ tiRNA ValCAC in the spinal cord of TDP43 mice. The results showed a significant (*P*=0.018) elevation of 5′ tiRNA ValCAC in the transgenic compared to non-transgenic mice (Fig. 7A). We also examined the levels of 5′ tiRNA LysCTT in the spinal cord samples of the SOD1 mutant and its respective non-transgenic controls and observed a trend towards increased 5′ tiRNA LysCTT expression in the mutant compared to its WT (Fig. S4).

Quantitative PCR (qPCR) analysis of 3′ tRF GlnCTG in the Tau model, 3′ tRF SerAGA in the TDP43 model and 3′ tRF CysGCA in the Tau model validated the downregulation of these tRFs in accordance with the RNA-seq analysis (Fig. 7B). Although the expression of 3′ tRF SerCGA in the SOD1 model by qPCR analysis did not match the RNA-seq analysis, its expression was consistent with the RNA-seq analysis in the TDP43 mouse model. The qPCR results for 3′ tRF ThrTGT and 3′ tRF TyrGTA from the TDP43 mouse model could not validate the results from the RNA-seq analysis.

### GO analysis reveals potential targets of 3′ tRF CysGCA involved in synaptic and neuronal functions

**Fig. 8.**
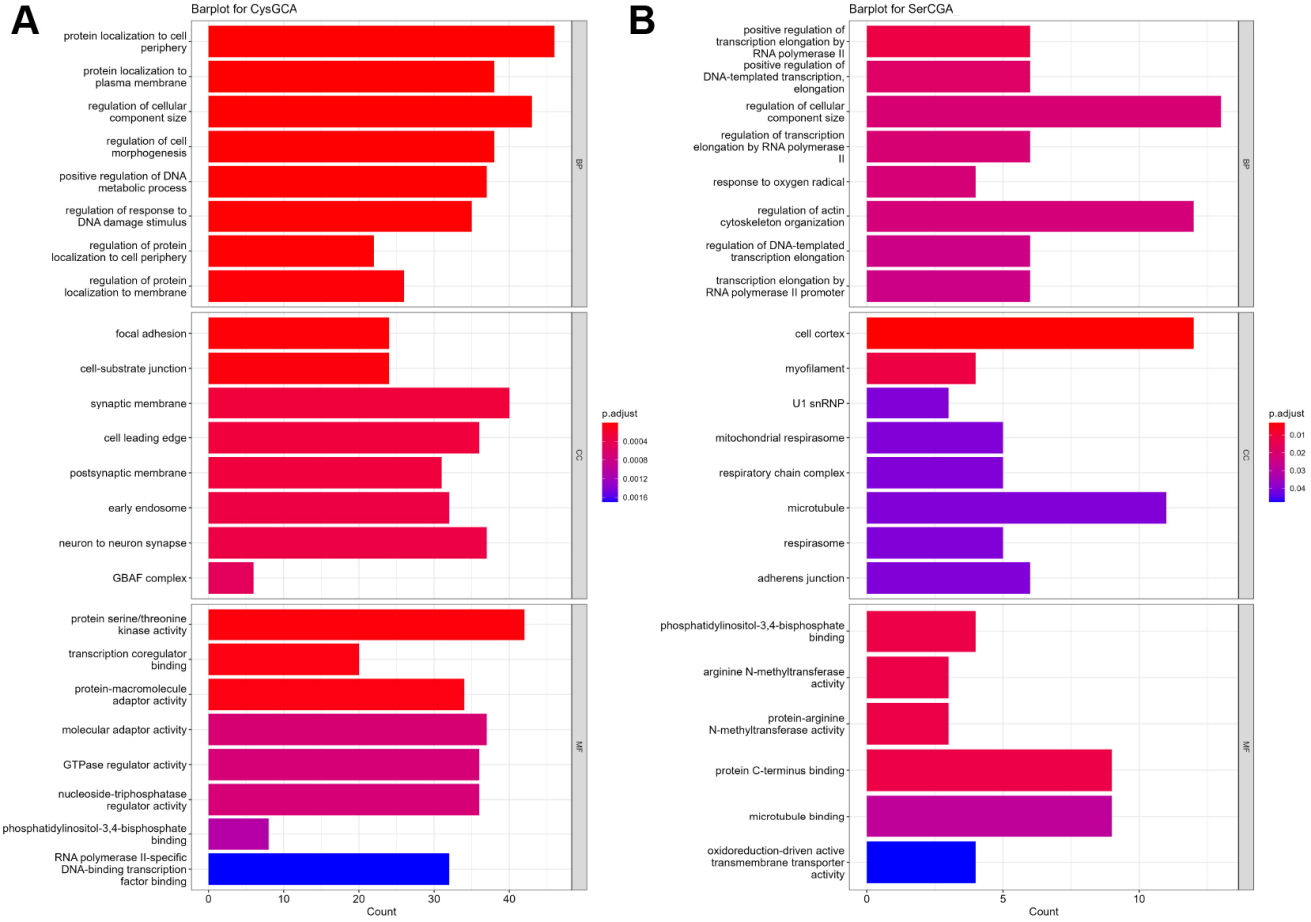
**GO analysis of potential targets identified for 3**′ **tRFs.** (A,B) Bar plots for Gene Ontology (GO) terms identified for potential targets regulated by 3′ tRF CysGCA (A) and 3′ tRF SerCGA (B), with the number of genes associated with each term in the *x*-axis and the GO terms for molecular function (MF), biological processes (BP) and cellular components (CC) on the *y*-axis. The colour of the bar represents the *P*-value. snRNP, small nuclear ribonucleoprotein.

We conducted Gene Ontology (GO) analysis of the targets identified by RNAhybrid for 3′ tRFs GlnCTG, SerAGA, CysGCA and SerCGA. The GO analysis of targets for GlnCTG and SerAGA did not reveal any statistically significant enrichments. The GO analysis of targets for 3′ tRF CysGCA, which was found to be downregulated in the Tau model, showed enrichment in protein localization to the cell periphery under biological processes. It also showed enrichment in synaptic membrane, postsynaptic membrane and neuron-to-neuron synapse in the cellular component domain, and transcription co-regulator and transcription factor binding in the molecular function category (Fig. 8A). The targets for 3′ tRF SerCGA, which was validated to be downregulated in the TDP43 mouse model, showed enrichment in transcription elongation in the biological processes category, myofilament and microtubule cellular components, and microtubule-binding molecular functions (Fig. 8B).

## DISCUSSION

ALS, FTD and PD, although being distinct NDs, exhibit shared genetic susceptibilities and pathologies. Here, we examined tsRNAs, a recently discovered class of sncRNA, to investigate whether the expression in these diseases could be correlated with the underlying disease mechanisms.

Isakova et al. (2020) investigated the different classes of sncRNAs in several tissues of mice and found distinct tissue-specific expression patterns of sncRNAs. Although the study found 5′ tiRNAs to be the most abundant in the brain, Haack et al. (2019) showed that the sncRNA expression and tRNA cleavage patterns varied across the different brain regions, including the amygdala, hippocampus, hypothalamus and adrenal gland. The authors demonstrated that the hippocampus has the lowest amounts of miRNAs compared to other brain regions (Haack et al., 2019). Additionally, 3′ tiRNAs emerged as the dominant class of tsRNAs in the hippocampus of pigs, suggesting a parallel trend in mammals. Conversely, Jehn et al. (2020) showed an abundance of miRNAs in the hippocampus of pigs. Despite contradictions arising owing to potential variations in tissue extraction methods, sequencing techniques and reference databases for genes, all studies consistently highlighted that the expression of sncRNA is tissue specific. This observation is mirrored in our study, where miRNAs emerge as the most prevalent class of sncRNA in the spinal cord, followed by tRNAs in both SOD1 and TDP43 models and their respective non-transgenic controls. However, tRNAs were found to be abundant in the hippocampus and substantia nigra, followed by miRNAs.

Following this, tsRNAs from glycine, glutamine, valine and lysine tRNAs have been shown to be the most abundant in different mouse tissues (Isakova et al., 2020). The same was observed in humans, and, additionally, tiRNAs generated by ANG cleavage were prominent (Thomas et al., 2018). Accordingly, we found tiRNAs from lysine and valine to be abundant in the samples from the spinal cord from SOD1 and TDP43 ALS mouse models, respectively. Moreover, 5′ tiRNA ValCAC was significantly elevated in both the spinal cord and serum of the SOD1^G93A^ ALS mouse model in the slow-progressing genetic background (C57) compared to its fast-progressing background (129Sv) (Hogg et al., 2020). Similarly, human serum samples from slow-progressing ALS patients exhibited higher 5′ tiRNA ValCAC levels compared to those from fast-progressing ALS patients, indicating potential prognostic value (Hogg et al., 2020). Joilin and co-workers also showed that 5′ tsRNA ValAAC is elevated in the serum of ALS patients compared to unaffected controls (Joilin et al., 2020). D'Erchia and colleagues also found an abundance of 5′ tiRNAs in the spinal cord of both ALS patients and controls. However, it was not differentially expressed, possibly due to the small sample size and patient heterogeneity (D'Erchia et al., 2017). Furthermore, when SOD1^G93A^ mice were injected with human ANG post-symptom onset, it delayed disease progression and increased lifespan (Crivello et al., 2018), demonstrating the protective role of ANG at the onset of ALS. Although the typical role of ANG is in the formation of blood vessels, with ribonuclease inhibitor (RNH1) inhibiting its ribonucleic activity in the nucleus, under stress conditions, ANG translocates to the stress granules formed in the cytosol and helps in the formation of tiRNAs unhindered by RNH1 (Yamasaki et al., 2009). Given that *ANG* mutations are causative of familial forms of ALS, PD and even Alzheimer's disease (Prehn and Jirström, 2020), defective action of ANG may be an underlying cause of impaired stress response and dysregulated tiRNA production. tsRNAs are predominantly hypomodified, indicating that they are derived from unmodified mature tRNAs (Pichot et al., 2023). However, tRNA modifications can also contribute to either increasing its susceptibility to ANG cleavage or providing protection against it. For example, loss of tRNA modifications such as cytosine-5 methylation caused by cytosine-5 tRNA methyltransferases such as DNA (cytosine-5-)-methyltransferase 2 (DNMT2) and NOP2/Sun RNA methyltransferase 2 (NSUN2) can increase vulnerability to stress and thus increase the production of tiRNAs by ANG cleavage (Kuhle et al., 2023). This suggests that although an elevated presence of tiRNAs indicates cellular stress, additional factors, such as a loss-of-function mutation in NSUN2 rendering the tRNA susceptible to ANG cleavage or a mutation in ANG itself, may be the underlying cause of aberrant tiRNA production.

We also observed lower expression of 3′ tRFs across all three neurodegenerative diseases. 3′ tRFs are products of tsRNA cleavage by DICER or other RNases. DICER, in particular, has been shown to be sensitive to cellular stress, leading to its downregulation (Garyali and Sengupta, 2019; De Cauwer et al., 2018; Anderson, 2012). Chmielarz and colleagues showed that DICER is downregulated in dopaminergic neurons of ageing mice, likely caused by prolonged cellular stress (Chmielarz et al., 2017). Moreover, downregulation of DICER has also been observed in ALS where miRNA expression was reduced owing to DICER protein interaction (Emde et al., 2015), and this might also lead to downregulation of 3′ tRFs. 3′ tRFs have been shown to load into AGO protein of the RNA-induced silencing complex and bind to mRNA, comparable to miRNA binding based on the sequence complementarity in the 6-8 nt seed region (Kumar et al., 2014). Dysregulation in the expression of 3′ tRFs has been shown to affect the expression of their target mRNAs (Kuscu et al., 2018). Although we found and validated four 3′ tRFs as being downregulated across the three diseases, 3′ tRF CysGCA downregulated in Tau mutant compared to its WT showed the most promising results in the GO analysis of its potential targets. 3′ tRF CysGCA has been shown to regulate the expression of JAK3 in chondrocytes under inflammatory stress by binding to AGO2 (Green et al., 2020). This implies that 3′ tRF CysGCA plays a role in gene silencing, and, given that the targets we identified were enriched in synaptic activity and presence of Tau at the synapse (Tracy et al., 2022), 3′ tRF CysGCA may be a promising biomarker for distinguishing Tau accumulation.

It is crucial to investigate the underlying mechanisms in NDs to develop potential diagnostic and prognostic biomarkers. In this study, we explored the landscape of tsRNAs in vulnerable tissues affected by ALS, FTD and PD to understand similarities and differences in disease mechanisms. Our findings demonstrate that sncRNA expression is tissue specific, and that the fragmentation of tsRNAs appears to exhibit both distinctiveness and commonality in the context of NDs. As tiRNAs generated by *ANG* are heightened in ALS, serving as a protective mechanism against stress, and considering the association of *ANG* mutations with ALS (Greenway et al., 2006), this might provide insights into the observed heterogeneity in disease progression and survival times. However, 3′ tRFs are downregulated in ALS and FTD, indicating a common mechanism for their generation. Furthermore, studies have shown that the presence of tsRNAs can be detected in blood samples from ALS patients (Joilin et al., 2020; Hogg et al., 2020). Therefore, validating the presence of these tsRNAs in serum samples from patients holds the potential to use them as circulating biomarkers. This could not only aid in diagnosing NDs but also help identify the specific endophenotypes at an early stage, enabling targeted treatments.

## MATERIALS AND METHODS

### Animal models

#### SOD1^G93A^ mouse model

Female transgenic SOD1^G93A^ mice bred on C57BL/6JOlaHsd (C57G93A) background, along with their non-transgenic female littermates, were employed for this investigation. These mice were kept in a controlled environment meeting specific pathogen-free standards, with conditions maintained at 22±1°C, 55±10% relative humidity and a 12 h light/dark cycle. They were housed in groups of three to four per cage and provided with standard pellet food (Altromin MT, Rieper, Bolzano, Italy) and water *ad libitum*. All procedures involving animal handling and welfare adhered to the institutional guidelines of the Mario Negri Institute for Pharmacological Research in Milan, Italy. These guidelines align with national regulations (D.lgs 26/2014; authorization no. 783/2016-PR issued on 8 August 2016 by the Ministry of Health) and internal policies of the Mario Negri Institute, including authorization for individuals conducting animal experiments (accredited under the quality management system certificate UNI EN ISO 9001:2008 reg. no. 6121). The study also conformed to the principles outlined in the National Institutes of Health Guide for the Care and Use of Laboratory Animals (2011 edition) and European Union (EU) directives and guidelines (EEC Council Directive 2010/63/UE). Animal protocols were approved by the Mario Negri Institute Animal Care and Use Committee and authorized by the Italian Ministerial Decree No. 246/2020-PR. Animals were deeply anaesthetized and euthanized at 18 weeks of age, corresponding to the symptom onset determined by the first sign of impaired paw grip strength and decline in body weight (Margotta et al., 2023). Mice were then transcardially perfused with PBS, and the spinal cord was dissected and frozen.

#### TDP43^A315T^ mouse model

All animal work was performed in accordance with the EU Directive (2010/63/EU) with ethical approval by the Royal College of Surgeons in Ireland (RCSI) Research Ethics Committee (REC1122), and under sequential licences from the Health Products Regulatory Authority (AE19127/P004 and AE19127/P054), Dublin, Ireland.

TDP43^A315T^ hemizygous mice on a congenic C57BL/6 background [B6.Cg-Tg (Prnp-TARDBP*A315T)95Balo/J], were purchased from The Jackson Laboratory (Bar Harbor, ME, USA) and originally generated in the laboratory of Dr Baloh (Wegorzewska et al., 2009). A TDP43^A315T^ colony was maintained on a high-fat jelly diet, which ensures the development of symptomatic motor dysfunction, and were monitored daily from postnatal day (PND) 80 for the development of symptoms (Coughlan et al., 2016). Only male mice were analysed, as female mice display variable disease penetrance (Hogg et al., 2018). The mice were genotyped by PCR and aged to specific time points for tissue collection; pre-symptomatic samples were collected at PND 60. Transgenic and non-transgenic littermates were housed in cages with three to five mice per cage, at constant temperature (22°C), on a 12 h light/dark cycle, with *ad libitum* access to food and water.

#### Tau^P301S^ mouse model

Transgenic Tau^P301S^ mice were bred and housed in the biomedical research facility at the RCSI. Tau^P301S^ mice overexpress the mutant (P301S) human *MAPT* gene encoding the T34 isoform of Tau (1N4R) under the mouse prion-protein promoter (Prnp) on a B6C3H/F1 genetic background (Yoshiyama et al., 2007). All animal experiments were performed in accordance with the principles of the European Communities Council Directive (2010/63/EU). Procedures were reviewed and approved by the Research Ethics Committee of the RCSI (REC201911006) and Health Products Regulatory Authority (AE19127/P056). All animals were treated according to European standards and regulations for animal experiments, and all efforts were made to minimize animal suffering and reduce the numbers of animals under experiments. Mice were housed in groups of two to five per cage and kept in a controlled animal facility on a 12 h light/dark cycle at 22±1°C and humidity of 40-60%. For our studies we used 6-month-old (pre-symptomatic) and 11-month-old (symptomatic) male heterozygous Tau^P301S^ mice and age-matched WT littermates. Mice were deeply anaesthetized and killed via cervical dislocation. Hippocampi were then removed and immediately frozen on dry ice and stored at −80C until further use.

#### Parkin/POLG mouse model

All animal work abided by the guidelines of the Canadian Council on Animal Care and was approved by the Animal Care Ethics Committee of the University of Ottawa. POLG^D257A/D257A^ mutator animals were obtained from The Jackson Laboratory [B6.129S7 (Cg)-*Polg^tm1Tprol^*/J] and previously characterized by Kujoth et al. (2005). The mutator mice were crossed with parkin knockout (KO) mice provided by Dr Michael Schlossmacher's laboratory (Ottawa Hospital Research Institute) (Itier, 2003). All mice were backcrossed to C57Bl/6 mice (Charles River Laboratories). The genotypes used in this study were WT (parkin WT; *Polg*^WT/WT^) and parkin KO; *Polg*^D257A/D257A^. Mice were given unlimited access to food and water. Both male and female mice were used. Animals were 12 months old at the time of the experiment to mark the start of dopaminergic neuronal loss modelling PD progression (Pickrell et al., 2015). Mice were genotyped with the following primer set: *Polg* F, 5′-TCCACTGAGGGAGCTTCTGT-3′; *Polg* R, 5′-CTTCCCTAAAGACCGCAGGG-3′; parkin WT F, 5′-TGCTCTGGGGTTCGTC-3′; parkin KO F, 5′-TTGTTTTGCCAAGTTCTAAT-3′; common R, 5′-TCCACTGGCAGAGTAAATGT-3′.

#### RNA extraction

Total RNA was extracted from lumbar spinal cord tissue of slow-progressing SOD1^G93A^ and TDP43^A315T^ mice and relative non-transgenic littermates using a Qiagen miRNeasy kit according to the manufacturer's instructions. RNA was eluted in 40 μl RNase-free water containing 1 μl RNaseOUT RNase inhibitor (Invitrogen). RNA purity and yield were analysed using a Nanodrop 2000 spectrophotometer (Thermo Fisher Scientific).

RNA was extracted from the hippocampus of Tau^P301S^ mice and relative non-transgenic littermates using the following protocol. The samples were placed in 1 ml Trizol and homogenized. For the phase separation, samples were incubated for 5 min at room temperature. We added 0.2 ml chloroform and inverted the tube ten times before incubating for 3 min at room temperature. The samples were centrifuged for 15 min at 12,000 ***g*** and 4°C, and the aqueous phase was transferred to a new tube. RNA was precipitated by adding 600 µl isopropanol to tubes and incubating for 10 min at room temperature. RNA wash was performed by removing the supernatant, washing the RNA pellet with 1 ml of 75% ethanol, vortexing briefly, centrifuging for 5 min at 7500 ***g*** and 4°C, and removing the ethanol. The pellets were air dried for 10 min. For solubilization, the pellets were resuspended in 20 µl RNase-free H_2_O and dissolved at 55°C for 5 min.

To extract RNA from parkin/POLG brains, we dissected a 1 mm punch of tissue from the substantia nigra pars compacta and flash froze the tissue in liquid nitrogen. Frozen tissue was thawed and ground using a motor-powered pestle in TRIzol (15596-026, Invitrogen) for RNA extraction.

### Small RNA-seq

Prior to library preparation, all RNA samples were treated with the demethylase AlkB. For each sample, 1 μg total RNA was prepared in the reaction buffer with 80 pmol purified AlkB in a total volume of 100 μl. The reaction buffer contained 300 mM KCl, 2 mM MgCl_2_, 50 μM (NH_4_)_2_Fe(SO_4_)_2_·6H_2_O, 300 μM 2-ketoglutarate, 2 mM L-ascorbic acid, 50 μg ml^−1^ bovine serum albumin, 50 mM MES buffer (pH 5.0). The reaction was incubated for 4 h at room temperature and quenched with 5 mM EDTA. Then, the treated RNA was recovered by RNA Clean & Concentrator Kits (R1013, Zymo Research). RNA samples from SOD1^G93A^, TDP43^A315T^ and Tau^P301S^ mouse models were prepared for small RNA-seq using a NEBNext^®^ Small RNA Library Prep kit (NEB). The parkin/POLG mouse model RNA samples were prepared using a QIAseq small RNA Library Prep kit (Qiagen). For all library preparations, a Pippin Prep (Sage Science) was used for size selection, allowing a library prepared from RNA up to 50 nt in length. This represents a larger than standard upper limit for small RNA-seq and is done to allow improved sequencing of tiRNAs. The finished libraries were quality controlled using an Agilent Bioanalyzer 2100. Libraries were sequenced on an Illumina sequencer. We generated an average of ∼4.6 million (M), ∼5.5 M, ∼44 M and ∼24 M reads per sample in the SOD1, TDP43, Tau and parkin/POLG mutant and non-transgenic models, respectively. The average per sequence quality scores were 34, 35, 39 and 35 for the SOD1, TDP43, Tau and parkin/POLG models, respectively, across the mutant and non-transgenic samples. Sequences of length less than 16 bp were removed, and quality control analysis showed that ∼3.6 M, ∼4.4 M, ∼34 M and ∼19 M of the total reads were retained from the SOD1, TDP43, Tau and parkin/POLG mouse models (Table S1). Of these reads, an average of ∼2.8 M, ∼3.3 M, ∼20 M and ∼11.9 M reads were mapped to the mouse genome in the mutant and non-transgenic samples for SOD1, TDP43, Tau and parkin/POLG models, respectively. These reads consisted of uniquely mapped reads and multi-mapped reads.

### Identification of tsRNAs

We used the Nextflow-based bioinformatics pipeline ‘tsRNAsearch’ (Donovan et al., 2021) to identify tsRNA fragments in the samples. tsRNAsearch takes raw fatsq reads as inputs and performs adapter trimming, maps to a custom ncRNA database and collapses the reads to generate the read counts for the tsRNAs. It then uses four methods to generate the depth files that contain the positional nucleotide read count. tsRNAsearch has implemented several strategies to counter the issues of reducing false positives when identifying tsRNAs, and detailed information can be found in the paper. The *P*-value generated by Fisher's method was used to calculate the adjusted *P*-value using Benjamini and Hochberg (1995).

We used the human protein atlas (Sjöstedt et al., 2020) to identify proteins expressed in the hippocampus and midbrain regions of mice. Next, we got the amino acid sequences for matching proteins retrieved from UniProt (UniProt protein ID UP000000589) (Bateman et al., 2023) and calculated the occurrence of each amino acid.

### Experimental validation of tiRNAs and tRFs

We performed qPCR validation of 5′ tiRNA LysCTT in the SOD1 spinal cord samples and 5′ tiRNA ValCAC in the TDP43 spinal cord samples using custom small RNA Taqman assays. The assays were designed to specifically detect 5′ LysCTT fragment (5′-GCCCGGCUAGCUCAGUCGGUAGAGCAUGAGACUC-3′) and 5′ ValCAC fragment (5′-GUUUCCGUAGUGUAGUGGUUAUCACGUUCGCCUC-3′). A detailed validation of the qPCR method can be found in our previous study (Hogg et al., 2020).

We further used a nrStar™ Mouse tRF PCR Array (AS-NR_002M-1, Arraystar, Rockville, MD, USA) to validate the tRF expression. All samples were prepped for the PCR array using a rtStar™ tRF&tiRNA Pretreatment Kit (AS-FS-005, Arraystar) and rtStar™ First-Strand cDNA Synthesis Kit (AS-FS-003, Arraystar). We utilized 1 µg RNA per sample for pre-treatment, which involved 3′-CP removal, 5′-P phosphorylation and a demethylation process. Subsequently, 100 ng of the pre-treated samples were utilized for cDNA synthesis, which includes a 3′ adaptor ligation, a 5′ adaptor ligation and the reverse transcription reaction. The qRT-PCR array was performed using Arraystar SYBR^®^ Green qPCR Master Mix (ROX-) (AS-MR-005-5), conforming to the manufacturer's protocol to amplify and quantify the cDNA. The results were analysed with the 2^−ΔΔCt^ method. The array identifies tRFs using the tRFdb nomenclature (Kumar et al., 2015). We mapped the tRFs identified in our study to the tRFs in tRFdb based on the length of the fragment and its position with respect to the tRNA.

### GO analysis of tRF targets

We used RNAhybrid (Kruger and Rehmsmeier, 2006) to identify the potential targets for the experimentally validated tRFs. RNAhybrid is used to calculate how well a short sequence hybridizes with a longer sequence and is primarily meant for miRNA target prediction. Studies have shown that many tRFs bind to AGO1, AGO3 and AGO4 and function similarly to miRNA in gene silencing (Kumar et al., 2014).

The inputs to RNAhybrid were protein-coding transcript sequences from Gencode release M33 (GRCm39) and tRF sequences for the tRFs validated by qPCR from tRFdb (Kumar et al., 2015). We set a minimum free energy threshold of −30 kcal mol^−1^ and *P*-value threshold of 0.01. R version 4.2.2 was used for all further analysis. The R package clusterProfiler (Yu et al., 2012) was used for GO analysis.

## Supplementary Material

10.1242/dmm.050870_sup1 Supplementary information

Table S1. Quality control metrics. List of samples, total sequences identified in the samples, filtered sequences and mapped sequences (both uniquely mapped and multimapped).

Table S2. tsRNAs identified in the study. List of tsRNA sequences, gene names and raw counts in each sample from the SOD1G93A vs WT, TDP43A315T vs WT, TauP301S vs WT and parkin/POLG vs WT comparisons.
