# Supplementary material for: Distinct fingerprints of tRNA-derived small non-coding RNA in animal models of neurodegeneration
Source: Dis Model Mech. 2024 Nov 18;17(11):dmm050870. doi: 10.1242/dmm.050870 (PMC11603119; doi:10.1242/dmm.050870)
Supplement: Supplementary information [file dmm-17-050870-s1.pdf]

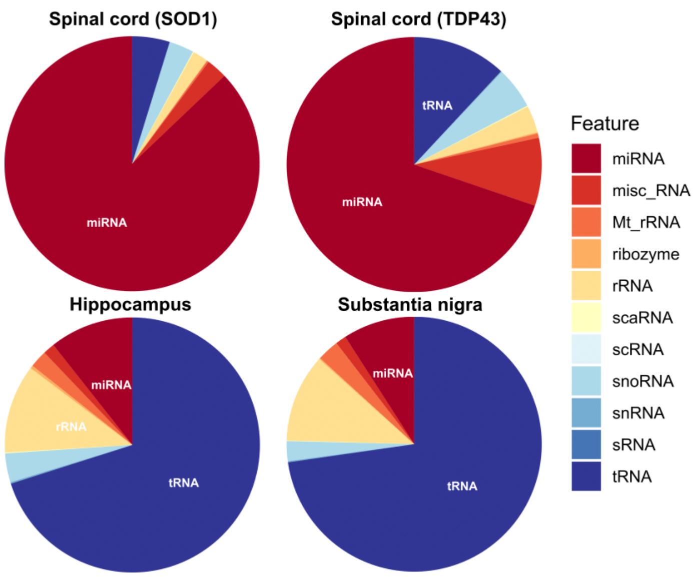

Fig. S1. sncRNA composition in different tissues of transgenic mice. Pie charts depicting the different classes of sncRNA. The colours represent different sncRNA class in mutant from spinal cord of SOD1 (n=4) and TDP43 (n=4) samples, hippocampus of Tau (n=4) samples and substantia nigra of parkin/POLG (n=2) samples.

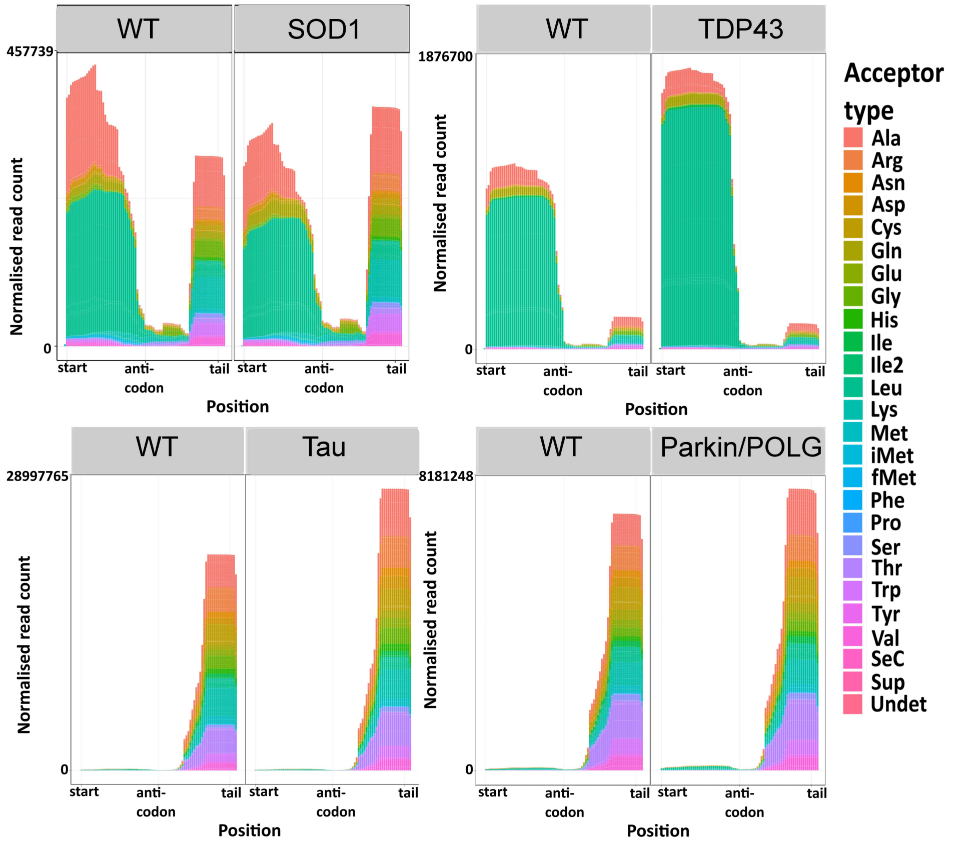

Fig. S2. tsRNA types and families identified in the study. tsRNA species identified by tRAX (Holmes et al., 2022) in SOD1 vs WT, TDP43 vs WT, Tau vs WT and parkin/POLG vs WT. The x-axis shows tRNA position including start (5' end), anticodon and tail end (3' end). The y-axis shows normalised read count.

Disease Models & Mechanisms • Supplementary information

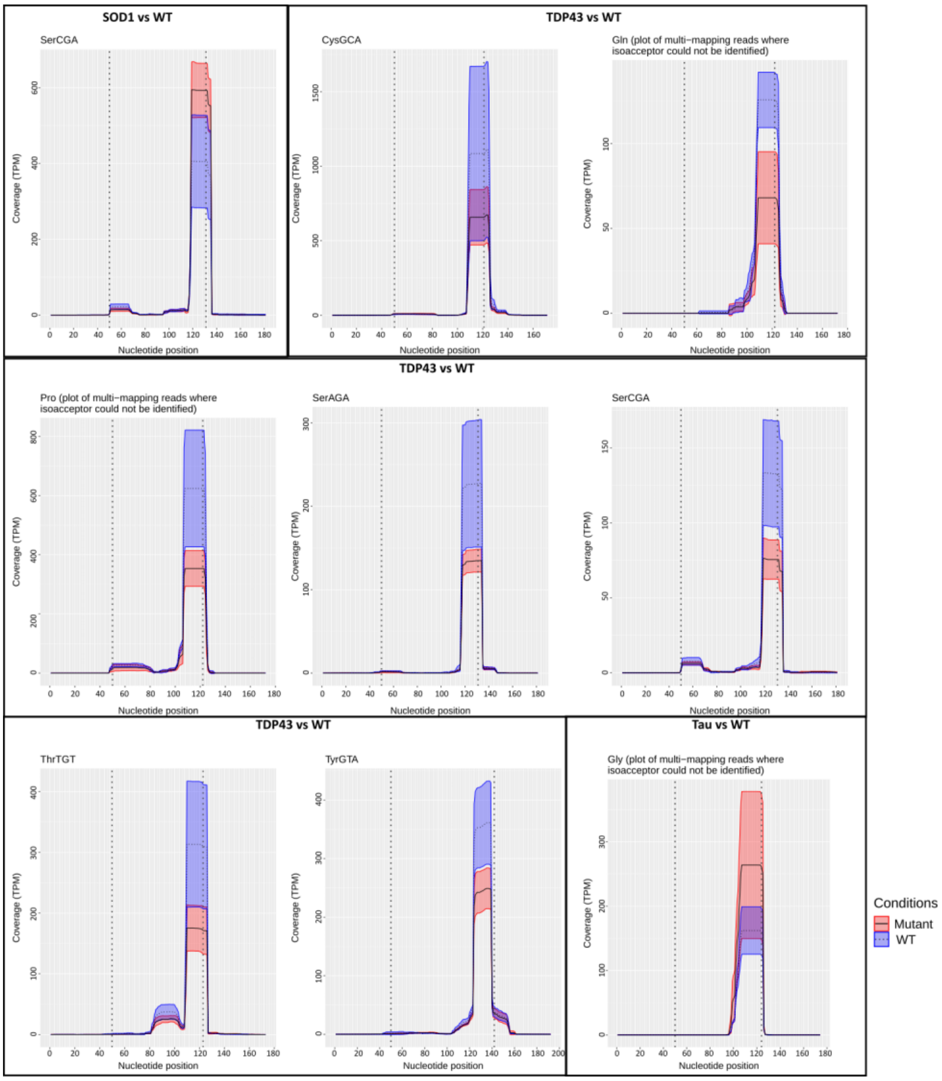

Fig. S3. Additional coverage plots of differentially expressed tsRNAs. Coverage plots of 3'tRF SerCGA in SOD1 (n=4) and its WT (n=4), 3'tRF CysGCA, 3'tRF Gln, 3'tRF Pro, 3'tRF SerAGA, 3'tRF SerCGA, 3'tRF ThrTGT and 3'tRF TyrGTA in TDP43 (n=4) and its WT (n=4), 3'tRF Gly in Tau (n=4) and its WT (n=3). The x-axis represents the nucleotide position and the y-axis represents the coverage in TPM. The two vertical dotted lines enclose the main tRNA segment of 70 nts, with an additional 50 nts included both upstream and downstream. The dotted line illustrates the mean coverage for WT samples, while the solid line represents the mean coverage for the mutant conditions including SOD1, TDP43, Tau, parkin/POLG, with the shaded areas indicating the standard deviation for both conditions.

Disease Models & Mechanisms • Supplementary information

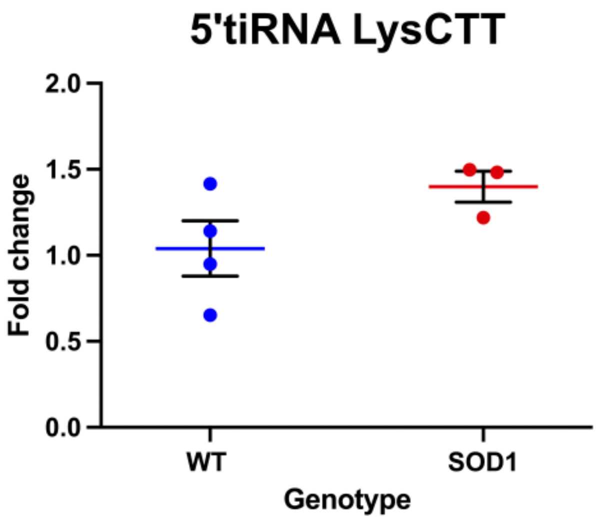

Fig. S4. qPCR validation of 5'tiRNA LysCTT. Levels of 5'tiRNA LysCTT in SOD1 and its WT quantified using a custom Taqman assay.

Table S1. Quality control metrics. List of samples, total sequences identified in the samples, filtered sequences and mapped sequences (both uniquely mapped and multimapped).

Available for download at
https://journals.biologists.com/dmm/article-lookup/doi/10.1242/dmm.050870#supplementary-data

Table S2. tsRNAs identified in the study. List of tsRNA sequences, gene names and raw counts in each sample from the SOD1G93A vs WT, TDP43A315T vs WT, TauP301S vs WT and parkin/POLG vs WT comparisons.

Available for download at
https://journals.biologists.com/dmm/article-lookup/doi/10.1242/dmm.050870#supplementary-data
